# On Corns and Other Things

**Published:** 1892-05

**Authors:** 


### ON CORNS, AND OTHER THINGS.

Corns are one of the most common and most persistent ills that afflict mankind; everybody has corns; and we never heard of a corn-afflicted individual who did not soak his feet and cut the corn with a sharp knife. This is the very best thing in the world to do, to make the corn hurt worse, and require longer to quit hurting. Corns should be trimmed *dry*, and with dull scissors. By a little care every particle of the callous tissue can be removed, and the part left soft and free from pain; but with a sharp knife one is apt to wound the soft part and produce inflammation. Then he has a sore toe in addition to the corn.

A corn is a singular illustration of how one's best intentions may be turned against him to his own hurt; (no pun intended). Corns are made by pressure of a hard substance on the flesh. This results in stimulating the epithelial layer of the epidermis into increased growth, proliferation, which, in our judgment, is clearly an effort on the part of the system to interpose an obstacle between the object pressing and the soft part pressed, for its protection; a corn is the the result, and instead of protecting the poor toe, it is worse afflicted. The *intention* on the part of the system is all right, no doubt; the vaso-motor system has charge of that department of the economy, and doubtless thinks it is doing the correct thing; but we can say of this as Wade Hampton is accused of having said of "good intentions," in general. —(You all know what he said.)

There is food here for reflection. This is one of the instances where it is clearly the doctor's course *not* to "assist nature," i. e., if her policy is to interpose a corn to prevent a pressure from hurting. The writer has often thought that the reason one substance will blister the skin and another will not is, that the nerves on guard at the extremities (the peripheral cutaneous nerve-filaments) either know, or have their instruction from the head-quarters, that certain substances, if allowed to come in contact with the skin will destroy it, and hence they turn out the garrison as it were, to put the invader out; or, in lieu of that, to interpose a bland fluid, the serum, between the destroyer and the destructible; clearly an effort to protect the skin, and they will *do it* even if the skin be destroyed in the attempt. But —if any one knows what will cure a *soft* corn, we are acquainted with an individual who will give him a dollar for the information.

Now don't think that individual is yours truly; never had a corn, and that is the reason he knows so much about them. He is like that party who wanted to borrow a dollar to get his book copyrighted. "What book have you written?" asked the other. "A book telling people how to get rich," said the author.

We know, that in light of modern research, the nervous system is, like a general in charge of troops, in authority over certain forces or elements whose duty clearly is to protect the system or any part, and that when invasion takes place at any point a contest for supremacy results. Why should this not explain the corn, as it explains the flow of tears when a cinder enters the eye, as an effort to remove it? We know the white corpuscles fight invading germs,—why not barricade to prevent damage at the toe portal? We see the same in the hands; from sweeping or using any hard substance callous forms; and if it is not to protect the soft skin—why it is done?

